# Composite lymphoma of T-cell rich, histiocyte-rich diffuse large B-cell lymphoma and nodular lymphocyte predominant Hodgkin lymphoma: a case report

**DOI:** 10.1186/s13256-021-02783-9

**Published:** 2021-04-14

**Authors:** Asil Esper, Sami Alhoulaiby, Ruba Zuhri Yafi, Zuheir Alshehabi

**Affiliations:** 1 Neurology department, Tishreen University hospital, Lattakia, Syria; 2 Faculty of Medicine, Damascus University, Damascus, Syria; 3 Faculty of Medicine, Damascus University, Damascus, Syria; 4 Pathology department, Tishreen University hospital, Lattakia, Syria

**Keywords:** Composite lymphoma, Hodgkin lymphoma, Diffuse large B-cell lymphoma, T-cell lymphoma

## Abstract

**Background:**

Composite lymphoma is a rare entity where two or more distinct subtypes of lymphoma coexist within a single organ or tissue.

**Case presentation:**

We report a new case of a 67-year-old Caucasian male patient, who presented with fatigue, weakness, weight loss, and polyuria. He also had epigastric and left lumbar pain, enlarged spleen, and enlarged left axillary lymph node on examination, with no relevant medical or familial history.

A biopsy from the node showed an appearance of T-cell rich, histiocyte-rich diffuse large B-cell lymphoma and nodular lymphocyte predominant Hodgkin lymphoma.

The patient was initially treated with adriamycin (doxorubicin), bleomycin, vinblastine, dacarbazine chemotherapy regimen, then switched to rituximab, cyclophosphamide, doxorubicin, vincristine, prednisone regimen.

During the therapy, some regression was noticed, especially in the size of the splenic enlargement; however, the patient died 2 months after completing the regimen.

**Conclusion:**

Composite lymphomas should continue to be studied. Also, treatment is still debatable in type, efficacy, and outcomes.

## Background

The term “composite lymphoma” (CL) was first used by Custer RP to describe the coexistence of more than one histological type of lymphoma in a single patient [1]; however, the present term is now limited to the rare coexistence of two or more morphologically and immunophenotypically distinct lymphoma clones occurring within a single organ or tissue [2]. Composite lymphoma incidence is low, varying from 1% to 4.7% [3].

The combination might include Hodgkin lymphoma (HL) with a B-cell or T-cell non-Hodgkin lymphoma (NHL), B-cell NHL with T-cell NHL, or two distinct B-cell or T-cell NHLs at the same anatomic site [2] 4.

We report a case of composite T-cell rich, histiocyte-rich diffuse large B-cell lymphoma (TCR-HR-DLBCL), and nodular lymphocyte predominant Hodgkin lymphoma (NLPHL) in the left axillary lymph node of a 67-year-old male from Syria.

## Case presentation

A 67-year-old Caucasian male presented to the department of gastroenterology at Tishreen University hospital, Lattakia, Syria, complaining of fatigue and weakness that began 1 month earlier accompanied by an unintentional weight loss of about 15 kg over a 15-day period.

Medical history includes hypertension and a cerebrovascular accident with no residual complications. The patient could not recollect the etiology of this accident. There was no history of familial lymphoma or cancer.

The patient had no vomiting, fever, or diarrhea but had polyuria and urine hesitancy, exertional dyspnea, and orthopnea.

Clinical examination revealed epigastric and left lumbar region tenderness with enlarged spleen during palpation, arthralgia, and enlarged hard left axillary lymph nodes. Vital parameters were pulse 100 beats per minute, blood pressure 110/70 mmHg, SpO2 95%, temperature 38°C. Positive laboratory tests included microcytic anemia (Hgb 7.6 g/dL, MCV 65.8 fL), decreased platelets (133 × 10^3^/µL), relatively decreased WBCs (4000 cells/µL), and increased ESR (69 mm/hour) (Table 1).

**Table 1:** Laboratory tests on admission

Test name	Result	Normal range
Complete blood count		
White blood cells	4000/μL	3500–10,000/μL
Neutrophils	2600/µL	1200–8000/µL
Lymphocytes	1000/µL	500–5000/µL
Red blood cells (RBCs)	3.4 × 10^6^/μL	3.5–5.5 × 10^6^/μL
Hemoglobin	7.6 g/dL	13–16 g/dL
Hematocrit	22.3%	38–53%
RBCs MCV	65.8 fL	82–96 fL
RBCs MCH	22.4 pg	27.5–33.2 pg
Platelets	133 × 10^3^/μL	150–450 × 10^3^/μL
Coagulation tests		
Prothrombin (PT)	65%	70–100%
Partial thromboplastin time (PTT)	28 seconds	20–38 seconds
International normalized ratio (INR)	1.32	1–1.5
Coombs direct	Negative	
Blood chemistry		
Urea	31 mg/dL	10–50 mg/dL
Creatinine	1.1 mg/dL	0.7–1.36 mg/dL
Total protein	4.7 g/dL	6.1–8.1 g/dL
Albumin	3.6 g/dL	3.5–5.2 g/dL
Aspartate aminotransferase (AST)	30 IU/L	8–2 IU/L
Alanine aminotransferase (ALT)	19 IU/L	8–20 IU/L
Lactate dehydrogenase (LDH)	1424 IU/L	50–150 IU/L
ESR	69 mm/hour	0–20 mm/hour
C-reactive protein (CRP)	64 mg/L	< 2 mg/L
Ferritin	418 μg/L	20–330 μg/L

**Fig. 1:**
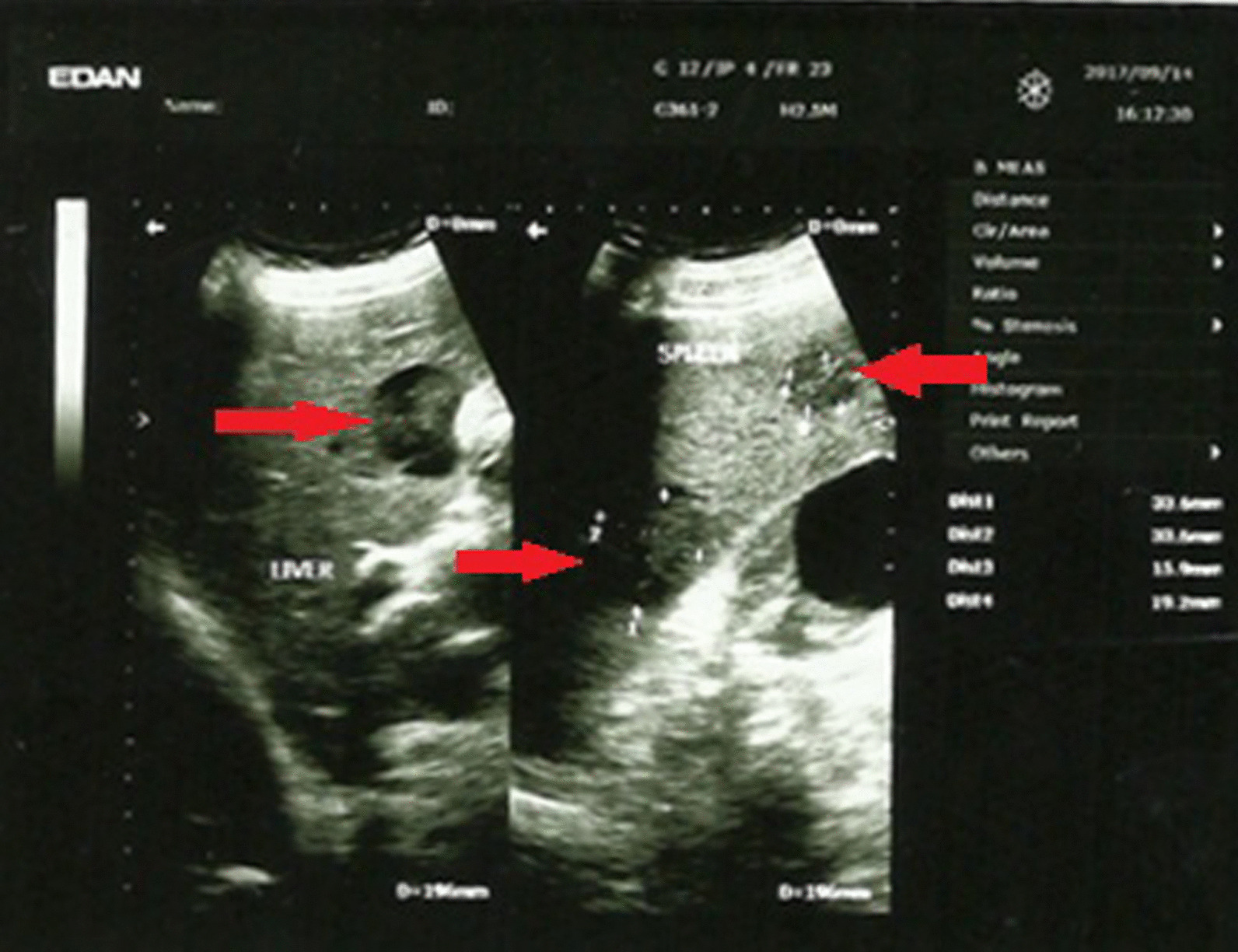
Ultrasound of the abdomen, showing a node in the liver (arrow, left) and intrasplenic nodes (arrows, right)

Ultrasound study of the abdomen and the pelvis showed a hypoechoic nodule in the liver (3 × 3 cm), a massive spleen enlargement (22 cm) with a few hypoechoic nodules, and a node above the splenic vein, possibly in the pancreas. The kidneys had a clear corticomedullary differentiation with a few simple cysts, the largest of which was (45 × 59 mm) in the left kidney and (67 × 78 mm) in the right one (Fig. 1). Computed tomography (CT) with intravenous (IV) contrast was then performed and showed homogeneous node measuring 35 mm in diameter in the fourth segment of the liver, homogeneous lobular splenic enlargement, bilateral cysts in the kidney with heterogeneous fixation of the contrast agent in the renal cortex, and calcifications in the coronary arteries, celiac trunk, and splenic artery. Based on investigations, lymphoma was suspected. Later, bone marrow aspiration showed normocellular, granular leukocytes in all stages of differentiation, increased eosinophils and neutrophils, plasma cells < 3%, and red blood cell precursors in all stages of differentiation without abnormalities. Megakaryocytes were normal in number with a slight decrease in size. The biopsy of the left axillary lymph node was then studied.

**Fig. 2:**
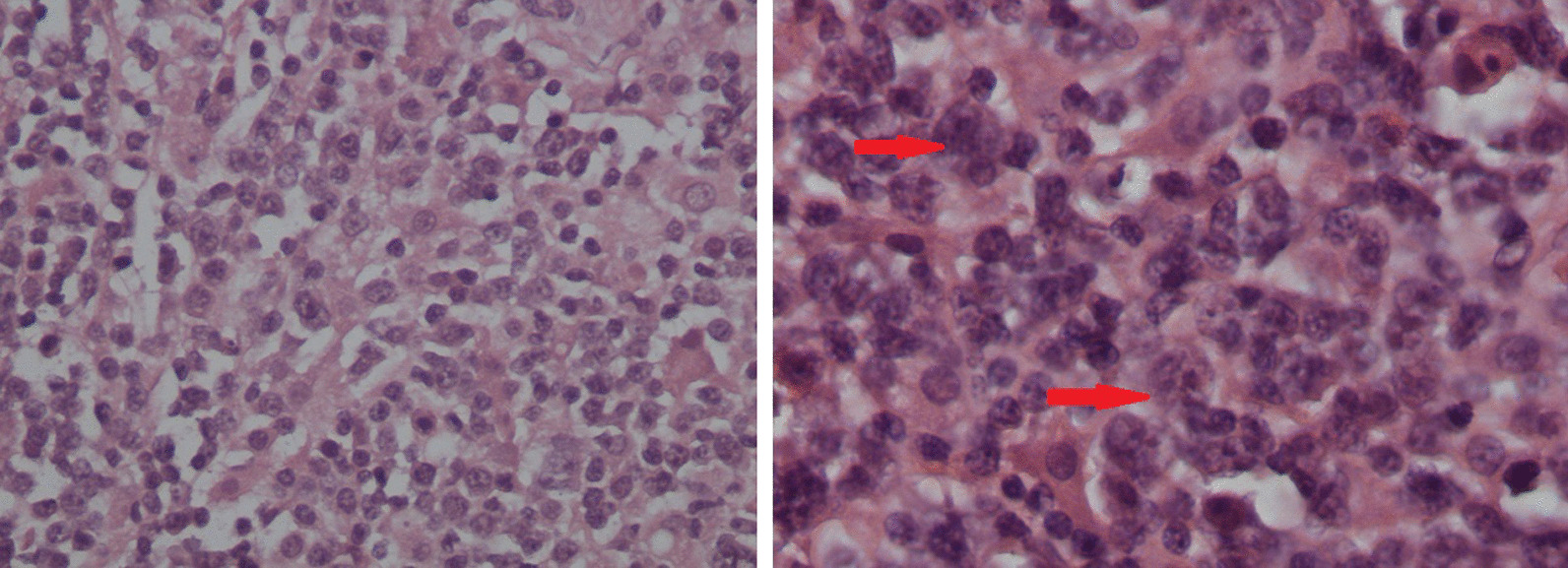
Pathology of the left axillary lymph node showing diffuse proliferation of large lymphoid cells (centroblasts and immunoblasts, arrows, right) admixed with small lymphoid cells, (hematoxylin and eosin [H&E] × 200)

**Fig. 3:**
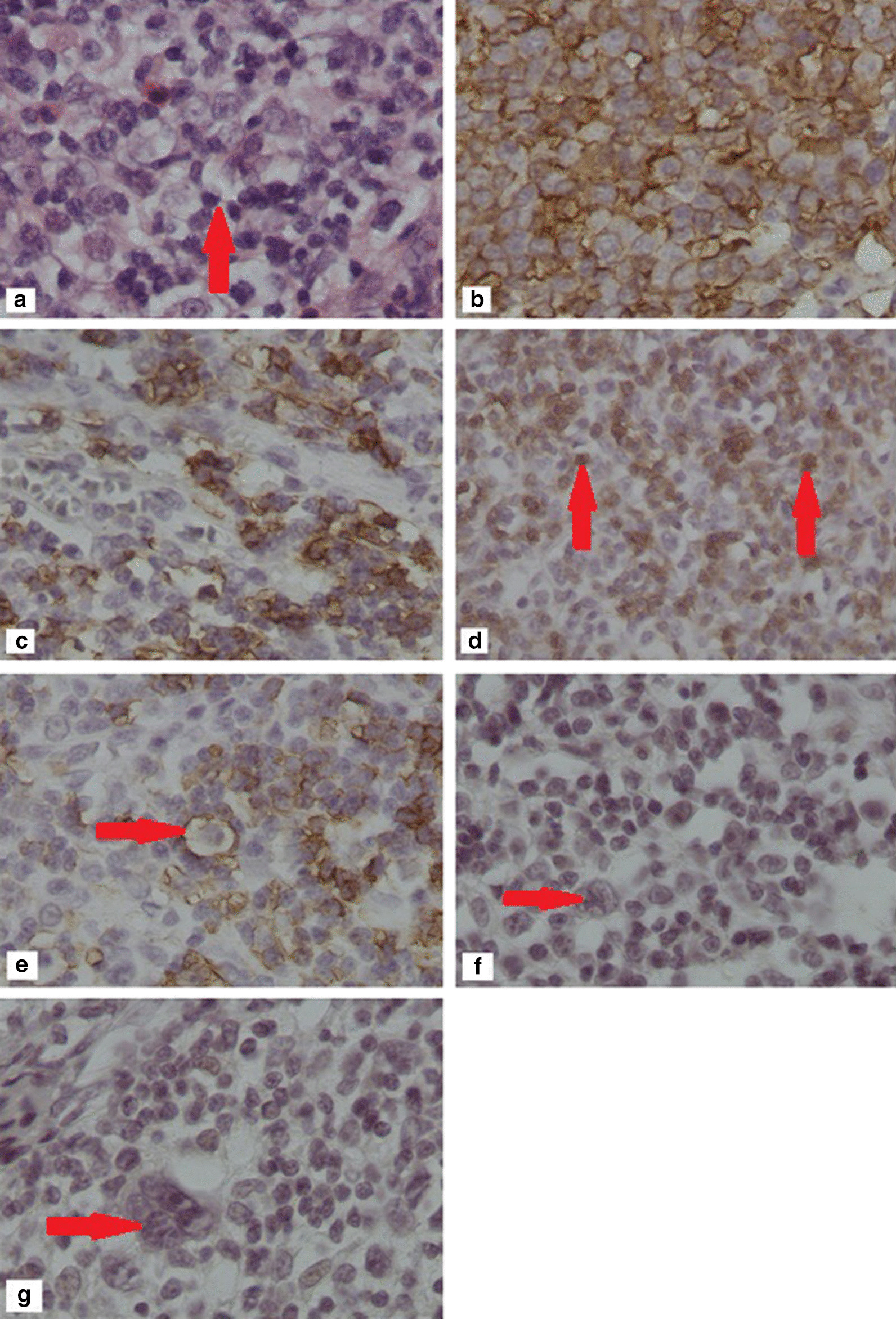
Pathology of the left axillary lymph node showing proliferation of Reed–Sternberg-like cells (Lymphocyte predominant cells LPs) admixed with mixed inflammatory cells (hematoxylin and eosin [H&E] × 400) [**a**, arrrow], CD20 positivity for large lymphoid cells × 400 [**b**], Bcl-2 positivity for large lymphoid cells × 200 [**c**], CD3 positivity for small lymphoid cells × 100 [**d**, arrows], LCA (CD45) positivity for LPs cells × 100 [**e**, arrow], CD30 negativity for LPs cells × 100 [**f**, arrow], and CD15 negativity for LPs × 200 [**g**, arrow]

Macroscopically, the lymph node was large (6 × 4 × 3 cm) and gray-tan in color with a soft consistency. Microscopy with the following immunostaining revealed foci with complete effacement of lymph node architecture and diffuse proliferation of cohesive large neoplastic lymphoid cells with large irregular nuclei and prominent nucleoli (Fig. 2). These cells were positive for CD20 and Bcl-2. The background cells were predominantly T lymphocytes (CD3+) and histiocytes, whereas B cells (CD20) were markedly depleted and Reed–Sternberg-like cells (LP cells) were absent. Other foci in the lymph node showed proliferation of LP cells with a background of mixed inflammatory exudates in the absence of CD20+ large lymphoid cells. LCA (CD45) and CD20 were positive for LP cells, whereas CD30 and CD15 were negative (Fig. 3). This panel supports the diagnosis of composite lymphoma.

**Fig. 4:**
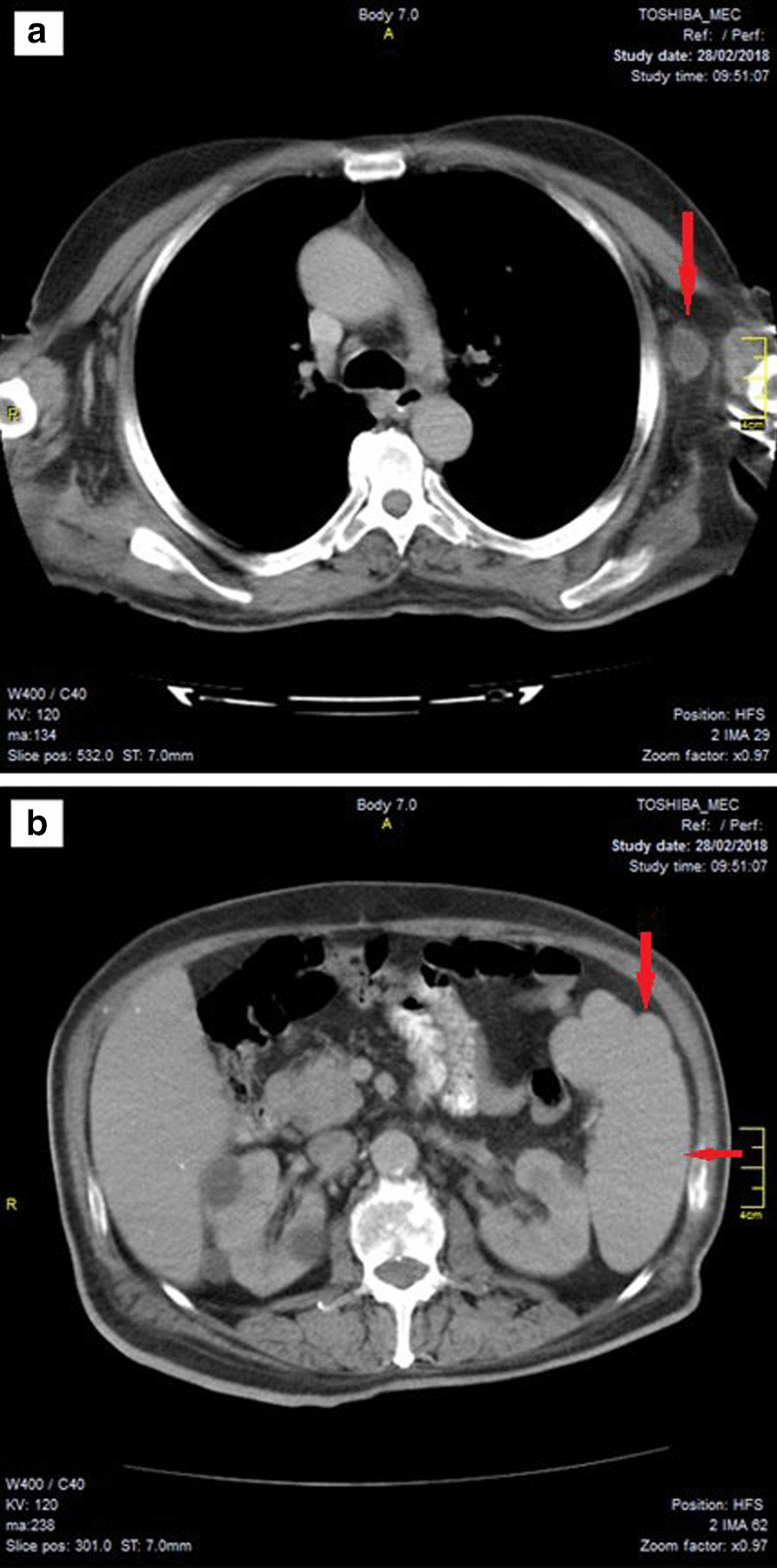
Computed tomography scan with IV contrast of the thorax and abdomen acquired 2 months after the initiation of treatment, showing left axillary nodule measuring 3.5 cm in diameter [**a**—arrow] and a mild splenic enlargement [**b**—arrows]

After the initial diagnosis of lymphoma, the patient was treated with once-every-2-weeks dose of adriamycin (doxorubicin), bleomycin, vinblastine, dacarbazine (ABVD) chemotherapy regimen for two sessions; then, he was switched to rituximab, cyclophosphamide, doxorubicin, vincristine, prednisone (R-CHOP) regimen after the diagnosis was confirmed with immunostaining and had 11 sessions every 3 weeks. Another CT scan with IV contrast was carried out 2 months after the initiation of the therapy, and it reported the presence of a left axillary nodule measuring 3.5 cm in diameter with smaller nodules in both axillae no more than (12 × 7 mm) in size. It also showed thickening in the stomach wall (up to 24 mm), in the cardia, and in the upper half of the body, as well as a mild splenic enlargement (Fig. 4).

The patient, unfortunately, died from cardiac arrest 2 months after the completion of the chemotherapy regimen. The patient’s family refused to perform an autopsy,  which prevented the establishment of an accurate causative relationship between the death and the disease.

## Discussion

Composite lymphoma was defined as the combination of more than one lymphoma in the same patient at different sites or in the same location. This definition has developed over time and currently is more accurately described as the presence of two or more types of lymphoma in the same lymph node or extranodal site. Usually one of these types is with low-grade follicular histologic characteristics and the other with diffuse architecture [4]. This entity is rare, occurring in about 1–4.7% of all lymphomas, and most of the reports are case reports or small case series. Cases that describe a composite lymphoma consisting of two or more types of NHL are more common than those reporting NHL and HL. Only six cases in the literature show a combination of classical Hodgkin lymphoma and DLBCL in the same site [2] 4, 5.

The diagnosis of composite lymphoma is based on diagnosing each lymphoma. TR-HR-DLCBL is diagnosed via pathologic appearance of DLBCL with less than 10% large neoplastic B cells on a ground of prominent inflammatory infiltrate of small T cells and histiocytes [6], [7]. NLP Hodgkin lymphoma affects a lymph node that is nodular, homogenous and pale, with no sclerosis between nodules (in comparison with classical HL), small B lymphocytes, and a variable number of large atypical cells with lobulated nuclei that resemble Reed–Sternberg cells (LP cells/popcorn cells) [7], 8. The immunohistochemical panel confirmed the diagnosis; however, both types exhibit almost the same panel [CD20+, CD45+, CD15–, CD30–, CD138–, and Bcl2 variable]. There are other tests that we could not perform on this patient [6]. It is worth mentioning that these two entities are possibly intertwined, as recent data suggest the possibility that NLPHL could proceed to/or contain areas of TR-HR-DLBCL [7]. In our case, the lymph node biopsy was interpreted as a composite lymphoma of TCR-HR-DLBCL and NLPHL, with the histoarchitecture of the lymph node and the composition of the background cell populations providing the most reliable diagnostic features for this case. The majority of NLPHLs demonstrate a nodular pattern. Our case demonstrates diffuse pattern, diffuse T-cell and histiocyte-rich infiltrate in the background. In conclusion, the unusual morphologic and immunophenotypic features of this challenging case support the diagnosis of composite lymphoma with features of TCR-HR-DLBCL & NLPHL rather than an NLPHL THRLBCL-like variant.

The etiology and pathogenesis of the different types of CL are not clear. Variable definitions and mechanisms have been proposed to explain this entity, of which the theory that the development of one type of lymphoma can induce the development of the other type is the most suitable [2]. Epstein–Barr virus (EBV) is suspected to cause composite lymphoma as it is known for multiple types of lymphoma, especially of B cells; however, proving the relation requires testing of p53 levels, which was not possible to perform.

The classification of composite lymphoma is still primitive, and the description of different combinations of lymphomas in the literature as non-Hodgkin lymphoma and Hodgkin lymphoma are B-cell lymphoma of any type and HL, T-cell lymphoma and HL, or even two distinct B-cell lymphomas [2].

Complete recovery is achieved in about 60% of patients with advanced disease on R-CHOP. In the series by Ho *et al.*, the first reported patient received six cycles of R-CHOP, initially achieving a complete response and subsequently relapsing with DLBCL 15 months after the completion of therapy. The second patient declined combination chemotherapy and was treated with single-agent rituximab, achieving stable disease [9]. ABVD is an effective choice in patients with Hodgkin lymphoma [10]. As it is a common older regimen, the physician used it after the initial diagnosis of the lymphoma. R-CHOP is found to be more effective than ABVD in non-Hodgkin lymphoma, as well as in advanced NLPHL, especially regarding the 10-year recurrence [10–12]; therefore, the patient was switched early to R-CHOP after immunostaining confirmed the presence of two types of lymphoma. After our patient received the R-CHOP chemotherapy regimen, he did not show firm evidence of response, with only confirmed regression in the size of the spleen, and he, unfortunately, died shortly after the end of the treatment.

## Conclusion

Composite lymphomas should continue to be studied, as morphology, etiology, and types of lymphomas contributing to the presentation vary. TCR-HR-DLBCL with NLPHL that we report is a valid variant. Treatment is still debatable in type and efficacy and the resulting quality of life, but R-CHOP regimen is a promising choice.

## Data Availability

All available data were incorporated into the production of this manuscript.

## Abbreviations

kg: Kilograms
SpO2: Saturation of peripheral oxygen
ESR: Erythrocyte sedimentation rate
Hgb: Hemoglobin
WBCs: White blood cells
MCV: Mean corpuscular volume
